# The impact of migration on the health status of Iranians: an integrative literature review

**DOI:** 10.1186/s12914-015-0058-7

**Published:** 2015-08-15

**Authors:** Sara Shishehgar, Leila Gholizadeh, Michelle DiGiacomo, Patricia M. Davidson

**Affiliations:** 1 Faculty of Health, University of Technology Sydney, Sydney, Australia; 2 Faculty of Health, Centre for Cardiovascular and Chronic Care, University of Technology Sydney, Sydney, Australia; 3 School of Nursing, Johns Hopkins University, Baltimore, MD USA

## Abstract

**Background:**

Immigration, both voluntary and forced, is driven by social, political and economic factors. Accordingly, some discussions and debates have emerged in recent years about the impact of migration on the health status of migrants. The aim of this review was to identify the impact of migration on the health status of Iranian immigrants and present a conceptual framework to facilitate the design and delivery of services and supports for this particular immigrant group.

**Methods:**

Data for this integrative review were collected from Medline, PsycINFO, Scopus, ProQuest, Academic Search Complete, CINAHL, and Google Scholar search engine. The database search was limited to peer-reviewed literature, published in English, between 1980 and 2013.

**Results:**

Twenty six articles were included in the review. Analysis revealed several factors influencing the mental health of immigrants, including language insufficiency; unemployment; sense of discrimination; cultural shock; lack of social support; lack of information about health care services; and intimate partner violence.

**Conclusion:**

Findings of this review have contributed to development of a conceptual framework that delineates the impact of migration on Iranian immigrants’ health. This conceptualization may also help in addressing the needs of other vulnerable groups during the transition phase of migration.

## What do we know?

- Migration can be a stressful experience.

## What does this paper add?

- This review outlines migration-related challenges that immigrants struggle with in order to integrate into host societies.
- Iranians’ view of health is different from Western concepts of health. As a consequence, Iranians’ socio-cultural values and beliefs should be taken into consideration in health care interactions.
- Despite negative effects, migration can have a positive impact on health and quality of life.
- Although immigrants may share similar experiences, social and cultural differences mean that Iranians may respond differently to migration challenges.

## Recommendations

- Further research should explore the socio-cultural values and challenges of migrants in host countries and examine how these affect mental health.
- Additional emphasis should be placed on understanding perspectives of vulnerable populations, such as refugee children, women, and the elderly.
- Using a strengths and resilience-based approach may be useful in intervention development.

## Introduction

Immigration, whether voluntary or forced, is increasingly driven by social, political and economic factors. As a consequence, some discussions and debates have emerged on the impact of migration on health status of immigrants [1, 2]. For generations, people have left their homelands and resettled in other countries seeking better future [3]. Such transitions can be challenging and may contribute to social marginalization, loss of social networks [4–6], health care access issues [7], and adverse health consequences, including depression and anxiety [8–11]. Not only do immigrants face challenges adapting to their new country, but many also continue to be impacted by the process of immigration, events that precipitated their departure, and ongoing relationships with friends and relatives in their home countries [12]. For example, events such as the Islamic revolution, political changes, war, and sanctions from the United States of America (USA) have compelled many Iranians to flee their homeland over the last thirty years [13, 14]. As a result, Iranians comprise a significant proportion of immigrants departing from the Middle East [14–18]. For example, the number of Iranian immigrants in Canada indicates a growth rate of 147 % from 1996 to 2006 [19]. In addition, Iranians can be found throughout the world such as Australia, Europe, Canada and Asia [20].

The increasing trends in migration worldwide have provided the impetus for focusing on the processes and outcomes of immigration. Yet, to date, there is limited research and information available that describes Iranian immigrants’ health status and migration outcomes [1, 2, 14, 21]. The available evidence suggests that Iranian immigrants are at risk of mental health problems. For example, the results of a study in Germany showed that 28 % of Iranian immigrants were suffering from mental disorders associated with acculturation stress [22], but further understanding of factors involved in succumbing to or preventing acculturation stress is unavailable. Without such an understanding, the needs of this group will remain unmet, leaving them vulnerable to adverse health and wellbeing outcomes in their new homelands.

Our aim in conducting this review was to ascertain information about Iranian immigrants’ resettlement experiences and health outcomes for the purpose of informing design and delivery of services and supports to prevent and reduce adverse effects of immigration. Although there has been much written about health outcomes of immigrants, this review will contribute the unique contextual experiences relevant to Iranian immigrants.

## Methods

### Search strategy

The search strategy was designed in consultation with a health librarian. Electronic databases searched were Medline, CINAHL, ProQuest, Academic Search Complete, Scopus, PsycINFO, and the Google Scholar search engine. Reference lists of the relevant literature were also reviewed for further related studies. Keywords used in the search were terms that depicted the person or event of migration (immigra* migrant*, emigrant*, exil*, refugee*), the target population (Iran*, Persia*) and health-related outcomes (health experience, health issue, health problem, mental health, psychological, mental problems).

### Selection of studies

Articles were included if they were written in English and published after 1980. This date was selected as it paralleled the first major wave of Iranian migration [14]. The review included studies of any design, involving adults (aged 18 years and over) who were Iranian immigrants departing their country for any reason, voluntary or forced and were settled in a host country. Articles were excluded if they did not focus on Iranian adult immigrants. Articles that focused on people from different nations were also included if they reported Iranians’ experiences separately when describing the results. Articles that focused on physical health issues without any consideration of the influence of immigration were excluded. This integrative review was guided by the principles of the Preferred Reporting Items for Systematic Review (PRISMA) [23]. Titles and abstracts of retrieved studies were reviewed to assess whether they met inclusion criteria. If inclusion was not immediately clear, full texts of articles were retrieved and reviewed.

### Data management and extraction

The first author extracted data from articles that met inclusion criteria and inserted information regarding aims, study design, sample size, geographic region of settlement, and outcomes into an excel spreadsheet to allow for tabulation and comparison across studies. A summary table was used to depict key themes and findings of included articles (Table 1). Discussions regarding data extraction were performed independently by two authors (SS-MD, SS-LG). Any disagreements were resolved through discussions until consensus reached.

**Table 1:** Included articles depicting Iranian immigrant studies

Author (year, Country)	Aim of study	Samples	Instruments	Main results	Type of study
Alizade-khoie 2011 Australia	To explore the impact of acculturation on health status	*N* = 302 Iranians Age > 65 y	Developed questionnaire from the NSW Older People’s Health Survey 1999	• Iranian elderly immigrants suffer from high level psychological issues and physical activity limitation	Quantitative
				• English proficiency decreases the rate of depression and anxiety	
Khavarpour 1997 [25] Australia	To determine the levels and predictors of psychological distress within the Iranians living in Sydney	*N* = 161 Iranians	General Health Questionnaire (GHQ-20)	• Students more likely to report psychological distress compared to full-time workers	Quantitative
				• migration contributes to psychological distress	
				• social support can reduce the experience of distress of unemployment and poor English proficiency	
Steel et al. 2011 [27] Australia	To examine for differences in the trajectory of psychological symptoms and key indices of social adaptation amongst refugees over two years	*N* = 104 Iranian and Afghan immigrant	• The Harvard trauma questionnaire	• Language insufficiency results in increasing mental distress, social isolation, difficulty in acculturation process, and on-going resettlement difficulties	Quantitative
			• The Hopkins symptom checklist-25		
			• The general health questionnaire		
			• The Penn State Worry questionnaire		
			• Post-migration living difficulties and detention experiences checklist		
Neale 2007 [33] Australia	To examine the knowledge, use and satisfaction of local health care services	*N* = 98 Iranians, Afghan and Iraqi *N* = 23 Iranians	• Semi structured questionnaire	• poor English proficiency = dissatisfaction from health care services	Qualitative
			• focus group		
			• multiple-choice questionnaire		
			•open-ended questionnaire		
Jafari 2010 [14] Canada	To examine the impact of immigration on mental health	*N* = 44 Iranians	• Focus group	• Low English proficiency resulted in social isolation, anxiety, mental problems, joblessness and unstable and aggressive behaviours	Qualitative
			• In-depth review		
Dastjerdi 2012 [3] Canada	To identify the obstacles and issues that Iranian immigrants face to access to health care services through the lens of Iranian health care providers	*N* = 50 Iranian immigrant who work as health providers	• in-depth semi-structured individual interviews	• Language barrier and lack of knowledge of Canadian health care systems.	Qualitative
			• three focus groups	• Lack of trust in Canadian health care services due to financial limitations and fear of disclosure	
			• Narrative inquiry		
Dastjerdi 2012 [15] Canada	To explore the Process of access to Health care services	*N* = 17 Iranians	• Individual face to face interview with a broad question then focused on health-relate experiences	• Getting isolated as a result of poor English skill	Qualitative
			• Telling story	• Tackling obstacles and being integrated	
Dossa 2002 [31] Canada	To explore the pedagogical potential of stories of post revolution Iranian women living in Canada	*N* = 40 Iranian women	• Semi-structured interview	• Iranians experience discrimination	Qualitative
			• two focus groups	• Iranians experience depression	
			• Story telling	• language barriers can result in unemployment or underemployment	
Tyndale et al. 2007 Canada	To explore the needs and experiences of Iranian immigrants about sexual health	*N* = 20 Iranians	• Semi structured interview	• difficulty in adjusting with new culture where sexuality is a usual fact	Qualitative
				• difficulties in receiving sexual health care because of misunderstanding (culture diversity) and shame and modesty	
Guruge 2012 [38] Canada	To examine the relationship of violence and physical and mental health	*N* = 30 Iranian women	• Brief symptom Inventory	• about one third of Iranian immigrant women suffer from mental illness because of intimate partner violence	Quantitative
			• Harvard trauma Questionnaire		
Ebrahimian 2012 Canada	To examine the effects of immigration on mental health of the Iranian immigrants residing in Toronto by comparing them to their counterparts in Iran	*N* = 200 Iranians	• Demographic questionnaire	• The rate of depression is higher amongst elderlies then younger immigrants	Quantitative
			• Depression Scale	• highly educated immigrants are less depressed than low-educated ones	
Singhammer 2011 [26] Denmark	To explore the relationship of violence and mental health among Iranian immigrants	*N* = 991 Iranian women	• A questionnaire including health indicators, health risk factors, healthy behaviours & health care services	• Iranian women had the greatest rate of divorce among other ethnic minorities in Denmark	Quantitative
				• The rate of violence was reported higher amongst Iranian women than other minorities	
Lipsicas et al. 2012 [4] European countries	To compare the frequencies of attempted suicide among immigrants and their hosts, between different immigrant groups, and between immigrants and their	*N* = 4160 immigrants from various countries included Iran	• Data were obtained from the WHO/EURO Multi-centre Study on Suicidal Behaviour	• Iranians displayed high suicide attempt rate in European countries despite low suicide rates in Iran	Quantitative
				• Immigration process in itself and the difficulties in acculturation can result in high- suicide attempt rates	
Haasen et al. 2008 [22] Germany	To find evidence for a relationship between acculturation stress and mental health problems, mainly depressive symptomatology	*N* = 100 Iranians	• Acculturation-stress-index (ASI)	• 28 % of Iranian immigrants suffer from mental disorders without treatment	Quantitative
			• SCL-90-R	• Depression score was high amongst Iranian immigrant	
			• Hamilton Depression scale (HAM-D)	• Inaccessibility of mental care centres	
Gerristen et al. 2006 Netherlands	To estimate the prevalence rates of physical and mental health	*N* = 410 Iranians, Afghan and Somali *N* = 117 Iranians	• medical outcome study (MOS)	• 43.4 % of Iranian asylum seekers suffer from depression and anxiety	Quantitative
			• SF-36		
			• Harvard trauma questionnaire	• Iranians suffer from dental and eye problems, back pain, neck/shoulder complaints, headache	
			• HSCL-25		
Akhavan 2007 [24] Sweden	To analyse females’ perceptions of various factors that influence their health	*N* = 10 Iranian women	• Semi-structured interview	• Discrimination is the greatest threat for health	Qualitative
				• Unemployment and financial issues result is mental problems	
				• Domestic violence, depression, and divorce as immigration adverse effects	
Bayard 2001 [34] Sweden	To examine the association between ethnicity among migrants born in Iran and psychiatric illness and intake of psychotropic drugs	*N* = 1980 Iranian, Kurd, Turkish, Polish, Chilean *N* = 293 Iranians	• Swedish Survey of Living Conditions questionnaire plus immigrant specific questions	• Iranian had more risk of mental illness and intake drugs 6 and 5fold more than swedes respectively.	Mixed(Qualitative and Quantitative)
			• Face to face interview	• Feeling discrimination by Iranians was higher than other ethnic minorities	
Momeni et al. 2011 Sweden	To investigate the self-reported mental health among two Iranian groups; in Sweden and Iran	*N* = 208 Iranians	• An author-made questionnaire	• 21 % of elder Iranian immigrants suffer from depression same as their counterparts in Iran	Quantitative
				• depression rate was higher among Iranian women compared to men	
Tinghog et al. 2010 Sweden	To investigate the association of immigrant and non-immigrant-specific factors with mental ill health within a diverse immigrant population	*N* = 720 from Iran, Iraq and Finland *N* = 250 Iranians	• The Hopkins symptom checklist-25	• 48 % of Iranian immigrants suffer from depression	Quantitative
			• The WHO (World Health Organization) Well-being Index	• 19 % of Iranian immigrants suffer from discrimination	
				• Unemployment and poor social network can lead to depression	
				• being female is a risk factor for mental disorders	
Wiking 2004 [36] Sweden	To analyse the association between ethnicity and poor health	*N* = 2160 From Poland, Iran and Turkey *N* = 480 Iranians	• Standardized & translated questionnaire for assessing the socioeconomic status (SES)	• Discrimination and acculturation are two important mediators between ethnicity and health.	Quantitative
				• High discrimination is felt by 34 % & 51 %, respectively, by men and women	
				• 41 % of women reported poor health status	
Lipson 1992 [28] The United States	To examine the immigration experiences of a sample of Iranians in the USA	*N* = 35 Iranians	• Semi-structured interview	• Lack of social support	Mixed(Qualitative and Quantitative)
			• Health opinion survey (HOS)	• Communication problems because of language insufficiency	
				• culture shock	
				• difficulty to find a good job	
				• Financial problems	
				• Ethnic bias (discrimination)	
Martin 2012 [37] The United States	To explore elderlies’ experience of discrimination in American health care system	*N* = 15 Iranians	• In-depth interview (in person)	• There was no discrimination	Qualitative
			• Open ended questions	• Highly positive impression of American health care providers	
				• Language barrier as a factor for underestimating possible discrimination	
Meleis et al. 1992 The United States	To investigate the nature of the relationship between demographic characteristics, ethnicity, length of time in the USA and physical and mental health/illness status, psychological well-being, and perceived health	*N* = 88 Egyptian, Yemeni, Iranian, Armenian, and Arab immigrant *N* = 16 Iranians	• Socio-demographic questionnaire	• unavailability of an ethnic community in overseas can result in depression and isolation among elderlies	Quantitative
			• Ethnic identity questionnaire		
			• 10-point rating scale	• Iranians usually enjoy from high integration and assimilation in host countries	
			• Cornell Medical Index (CMI)		
			• Revised Bradburn Morale Scale	• integration increases along with increasing the length of stay in the host country	
			• 10-point Cantril ladder scale	• increasing the length of stay in the host country doesn’t improve the immigrants’ health situation	
Saechao et al. 2012 [29] The United States	To examine stressors and barriers to using mental health services among first-generation	*N* = 30 from Cambodia, Iran, Iraq, Vietnam, Africa, eastern European *N* = 4 Iranians	• Six focus groups	• Barriers: Language, cost, lack of information about mental health services	Qualitative
				• Stressors: discrimination, economic status, difficulty to find suitable job	
Ghaffarian 1998 The United States	To explore the relationship of acculturation and mental health	*N* = 238 Iranians	A five section questionnaire including:	• Acculturation increased = score of mental health decreased (better)	Quantitative
			• Demographic Questions	• Men are healthier than women mentally	
			• Warheit & Buhl's Anxiety, depression and Psychological dysfunction scale		
			• Iranian version of Mendoza ‘s Cultural Life Style Inventory		
Ghaffarian 1987 The United States	To examine Iranian immigrants, their acculturation to the American culture, and specifically, the acculturative differences between males and females	*N* = 110 Iranians	• Demographic Questionnaire	• Less adjustment to host culture = stress and depression	Quantitative
			• Warheit & Buhl's Anxiety scale	• Men are more able to adjust themselves with new societies and cultures	
			• Traditional family ideology designed by Levinson and Huffman (1955)		
			• Acculturation scale designed by Cuellar, Harris, and Jasso (1980)		

### Methodological assessment and data analysis

The first author assessed quality of the included articles using Critical Appraisal Skills Program (CASP). The studies were separated into qualitative, quantitative and mixed-method studies. A general inductive analysis approach was used to derive themes from the findings. Where multiple nationalities were included, results depicting only Iranian participants were extracted. Extracted themes and inconsistencies were discussed among the authors.

## Results

**Fig. 1:**
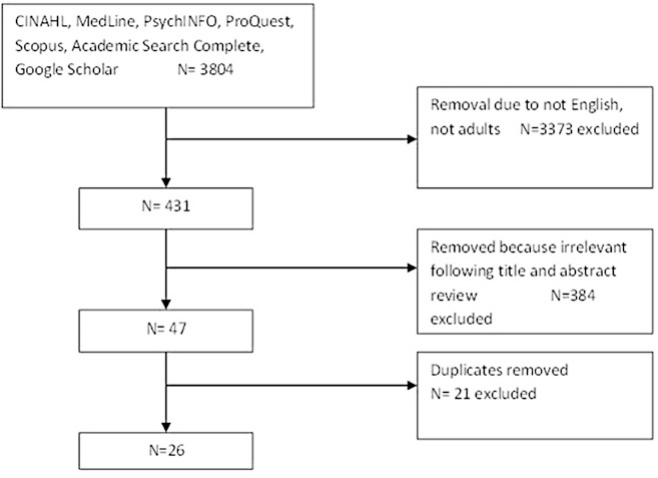
PRISMA flow chart depicting study selection

**Fig. 2:**
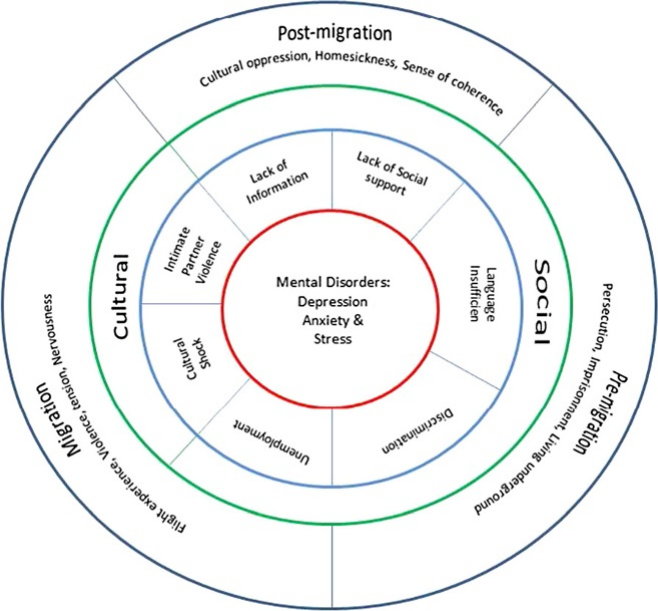
Framework of concepts pertinent to Iranian immigrant experiences

Following application of inclusion and exclusion criteria and removal of duplicates, 26 articles were included in the review (Fig. 1). Of these, sixteen studies were quantitative, nine were qualitative and two used mixed-methods (Table 1). Data collection methods included focus groups and interviews with Iranian immigrants, cross-sectional surveys, and randomised control trials. Qualitative studies depicted the experiences and their relationship with immigrants’ health status. Quantitative studies mainly centred on prevalence of negative experiences and their association with mental disorders. All included studies were conducted in Western countries such as Sweden, Canada, and the USA. Themes derived from the articles reflected the socio-cultural lens of migration in respect of phases of transition: including pre-migration, migration and post-migration (Fig. 2).

### Qualitative studies

Data were collected through face-to-face interviews and focus groups. Importantly, maintaining anonymity and confidentiality were paramount ethical considerations involved in conducting research with these populations. Audio recording was refused by participants in one study because of their previous experiences of information gathering for political purposes and resultant distrust [24]. Despite such risks, only eleven studies out of 26 provided explicit statements regarding ethical considerations.

### Quantitative studies

Cross-sectional data were collected predominantly *via* close-ended questionnaires administered *via* face-to-face interviews. Two studies asked respondents to complete questionnaires using web-based survey platforms or *via* telephone interview [25, 26].

Findings of the included studies revealed seven sub-themes under two main categories of social and cultural issues, that represent common problems faced by Iranian immigrants during and after immigration and throughout the settlement process. The main themes and associated sub-themes are presented in Fig. 2 and explained in the following section.

### Social issues

#### Language barriers

Learning the host country language is an important factor in social engagement. It seems that inability to understand English affects all aspects of Iranian immigrant life. Poor language skills has been related to communication difficulties, protracted resettlement and acculturation processes, and mental distress [27]. The adverse experiences may contribute to social isolation, anxiety and depression [3, 27]. Just as language proficiency can affect immigrants’ health, immigrants’ wellbeing can influence their language acquisition. Impaired mental health can challenge one’s adjustment and language acquisition [27]. Steel et al.’s study [27] found that refugees with permanent protection visas had higher language acquisition compared to refugees with temporary protection visas [27]. Participants with temporary protection visas showed no significant improvement in their language skills over time, leading to increase the rate of mental distress, depression, and social isolation in this population. In contrast, those with permanent protection visas demonstrated a significant improvement in their language ability, engaged in social activities, and integrated well into the host society [27]. Together, these data exhibit a pattern of increased mental distress amongst immigrants with low levels of host language proficiency.

#### Employment

Inability to find employment commensurate with qualifications was identified as an important stressor that Iranians experienced in their new countries. Underemployment was reported to lead to insecure economic conditions, high stress levels, depression, anxiety, and other mental disorders [14, 28–30]. Underemployment and unemployment contribute to low self-esteem and self-confidence in Iranian immigrants, who were previously proud of their prominent employment roles in their home country [24]. Lipson [28] highlighted that even highly skilled Iranian professionals could not find a suitable work upon arrival to the USA. The value of employment was exemplified by Iranian women in Canada, who conveyed that a meaningful occupation can mitigate painful experiences of immigration, such as separation from their children [14, 31].

#### Lack of information about health care services

Limited knowledge of health care services is another obstacle that Iranian immigrants often faced upon arrival to their host countries. Lack of awareness of health care services can delay and inhibit health care-seeking activities, and is likely exacerbate existing conditions [32]. In some countries such as Canada, immigrants are provided with many forms and pamphlets about daily living needs including information about available health services. In some cases, these resources have been described by Iranian immigrants as being too long and difficult to understand [32].

Access to health care services is critical in addressing mental health problems of immigrants. Results of a study showed that approximately 28 % of Iranian immigrants living in Germany were suffering from untreated mental disorders because their lack of access to appropriate mental health services [22]. Likewise, Neale et al. [33] identified that confusion and lack of information about the Australian health care system resulted in increased mental problems for immigrants.

#### Lack of social support

Leaving friends and families behind during the immigration process is a painful, yet common experience. Lack of social support can negatively affect individuals’ health [28]. While, support from family and friends and a social network may mitigate the adverse impacts of immigration-related stressors, such as unemployment and poor language proficiency [25]. Lipson [28] described Iranians as a multi-cultural, multi-language, and a multi-religion population, and therefore, they were not frequently part of a cohesive homogenous social network. In contrast, a study in Sweden found that 72 % of Iranians had a social network; however, the remaining subset (28 %) reported poor social networks and social support and associated mental health problems [30].

#### Sense of discrimination

Iranian immigrants reported experiencing discrimination. Ten of the 26 studies considered discrimination to be a significant factor leading to depression and mental disorders [24, 28–31, 34–37]. In a study, 59.6 % of Iranian immigrants living in Sweden had perceived ethnic discrimination [30]. Similarly, Wiking et al.’s study [36] in Sweden found that 34 and 51 % of Iranian men and women, respectively, experienced discrimination when using health care services. However, Martin [37] did not report any forms of discrimination against this ethnic minority by health care providers and physicians in the USA. Yet, discrimination in educational centres, such as schools and English language courses has been reported by Iranian immigrants in studies that were carried out in the USA and Canada [29, 31]. Some immigrants felt they were judged negatively on because of their religion and accent [29].

### Cultural issues

#### Culture shock

Culture shock is defined as diversities in expectations, values, and social norms that might be experienced by immigrants in western countries either in their social communications although they may not react effectively to this problem [28, 34, 35]. Divergent cultural norms can result in conflict between parents and children, child-rearing styles, relationship breakdown, and divorce [14, 28]. Inability to adjust to cultural differences can contribute to depression in Iranians [36]. Cultural differences can also influence immigrants’ health seeking behaviours. Some immigrants experience numerous communication problems, not only because of their English language deficiencies, but as a result of cultural misunderstandings wherein health providers misinterpret, immigrants’ discomfort or distress [28]. Another cultural difference between western countries and Iran relates to sexual content in the media and community. Many Iranian women in these countries are concerned about the effects of these exposures on their relationship with their husband and resultant expectations [37].

#### Intimate partner violence

Violence by intimate partner was reported in three of the 26 articles [24, 26, 38]. Violent behaviours may include being kicked, slapped, dragged, shoved, forced to have sexual intercourse, beaten, and restricted from attending social activities [38]. An Iranian woman in Sweden reported that her husband did not allow her to go to work or attend classes. Consequently, she divorced him to maintain her dignity and mental wellbeing [24]. Although several studies found that exposure to family violence was strongly associated with self-reported mental health problems of Iranian immigrants [24, 26], Guruge et al.’s study [38] failed to find a significant relationship between health status and exposure to violent behaviours among this immigrant population.

## Discussion

In this review, we have highlighted the challenges that Iranian immigrants encounter during resettlement in host countries, and discussed the impact of associated negative experiences on their health and wellbeing. The results of this review revealed that immigration may contribute to adverse psychological outcomes. These data contributed to development of a conceptual framework that addresses the main challenges faced by Iranian immigrants across pre-during-post migration phases and how these experiences affect the immigrants’ mental health, including experience of stress, anxiety, and depression (Fig. 2).

The conceptual framework reflects social and cultural issues contributing to mental health problems among this immigrant population group. Social issues, including experience of discrimination, language barriers, lack of information about health care services, lack of social support, and unemployment can have adverse effects on immigrants’ health. Similarly, cultural issues including intimate partner violence and culture shock increase their risk of developing physiological problems. These key factors are discussed in relation to the health of immigrants.

The challenges identified in the literature appear relevant to many immigrant populations, however, Iranian immigrants are likely to be particularly at higher for mental disorders. Pre migration experiences, such as the Islamic revolution of Iran, the eight-year Iran-Iraq War, and the recent economic sanctions against this country can negatively affect Iranians’ mental health.

The findings of this review also suggest that language barriers hinder effective communication of immigrants with mainstream communities, leading to social isolation, and lack of utilisation of social services, including health care services [3, 14, 27–29, 31, 33, 40, 43]. These negative experiences have been linked to exacerbation of mental health problems in this population group [44]. Yet, health care workers do not perceive linguistic limitations as a barrier to the use of health care services and poor health status of immigrants [32]. From the point of view of health providers, cultural misunderstanding and lack of awareness of health care services are more important factors that can result in dissatisfaction with health care systems rather than language insufficiency [32]. Another migration-related factor which influences the health of immigrants is their employment status. Almost all studies in this review asserted that unemployment and underemployment were common challenges that Iranian immigrants endured [14, 24, 28–31]. These studies depicted the negative effects of unemployment on mental health of immigrants, such as reduced self-esteem and self-confidence and high levels of stress, anxiety, and depression. Unemployment is particularly problematic for Iranian immigrant compared to other Middle Eastern immigrants, as they are more likely to be highly educated and possess high social standing in their origin country. The inverse relationship between education and employment has contributed to poor mental health outcomes among Iranian immigrants [26, 45].

Generally, immigrants report lack of social support in a new country. While Iranians have been observed as a well-organised community in Sweden [30], another study reported that Iranians do not develop a cohesive organised community in the USA [28]. This was partially related to the existing diversities in Iranian’s culture, religions and political and economic issues, which are often carried forward into immigrants’ new life [46]. The inconsistency in the findings may be a result of different methodologies employed, timing, and settings of the studies. Further, the political climate that characterises different time periods possibly contributed to the immigrants’ socialisation and their congregation behaviours. For example, the Islamic revolution of Iran, and the resultant political unrest may have influenced Iranians’ behaviours at that time, resulting in limited trust and unitedness among Iranian immigrants. After several decades of political conflict, however, Iranians may have decided to become more united to be able to help themselves and fellow immigrants in a new country. Evidence reveals a direct relationship between lack of social support and mental disorders [28, 30], yet, social support cannot guarantee mental wellbeing [47].

Many studies have reported the experience of different types of discriminations by Iranian immigrants, and how these negative social experiences affected different aspects of the immigrants’ life, particularly their mental health [24, 30, 31, 39]. It is argued that discrimination towards immigrants is likely to be underestimated due to language and cultural differences [48]. The media’s negative portrayal of Islam and Iran is likely has contributed to the public’s perceptions about migrants from Middle East and their discriminative behaviours. Discrimination can lead to mental health disorders, reduced self-confidence, and social isolation, making acculturation and resettlement more difficult for immigrants [40].

Apart from the social challenges, exposure to a new culture and new ways of living can be the source of considerable dissonance among family members, affecting their relationships and expectations of each other. Iranian immigrants have been recognised as people who are willing to integrate with host cultures, but they also do not like to give up their customs [45]. In other words, Iranians carry their ‘cultural baggage’ as well as demographic profiles wherever they go [14, 36]. Intimate partner violence is likely to be intensified by migration processes and the related stresses, increasing the risk of developing mental health problems such as anxiety and depression among immigrants [24, 26]. Guruge et al. [38], however, did not find a significant relationship between intimate partner violence and mental disorders [42]. This study failed to provide an explanation for the inconsistent finding. The small sample sizes of the relevant studies may account for the inconsistency in the findings. Studies with larger sample sizes would be necessary to help generalise the results to the wider community. Overall, the findings of this review suggest that Iranian immigrants are at higher risk of developing mental health problems. While mental health is viewed as part of overall health in Iranians’ culture and medicine [39], the considerable cultural stigma towards mental illnesses may hinder the use of mental health services for Iranian immigrants and can hinder seek of mental health services [14].

The literature on immigrant has mainly focused on negative outcomes of immigration, and overall immigrants have been portrayed in the literature as ‘victims’ in immigration process, however, Sulaiman-Hills and Thompson (2012) in their study on Kurdish and Afghan refugees in Western Australia and New Zealand established a new perspective on immigration. They found that migration could provide new education and occupational opportunities for immigrant women [49]. In line with this finding, evidence suggests that gender plays a role in mental health of immigrants [26, 36]. How the role of gender in resettlement process and mental wellbeing of Iranian immigrants is still controversial. While some studies suggest that Iranian men have a higher level of acculturation and superior mental health compared to Iranian women [41, 50, 51], Moghissi (1999) found that compared to men, Iranian women were healthier mentally and could better integrate into Canada’s society. This finding was justified by the fact that Iranian women are used to accepting changes and adjusting to changes due to sociocultural factors. For example, many Iranian women have to live with their husband’s family despite their divergent attitudes and culture. Though these experiences Iranian woman learn strategies to cope with new changes [41].

Overall, there is a shortage of studies focusing on Iranian immigrants and their mental health issues, likely due to the difficulties in conducting research on minorities. Possible positive outcomes of immigration, such as freedom, living in a ‘well-organized’ society, greater facilities, and support of government, need to be explored by research, particularly from immigrant women’s perspectives [49].

## Conclusion

The conceptual framework derived from this integrative review suggest that mental health of Iran immigrants can be affected by the challenges that their encounter across pre, during, and post phases of migration. Pre migration stresses, language barriers, unemployment, lack of information about health services, social isolation, experience of discrimination, cultural shock as well as intimate partner violence can adversely affect wellbeing and mental health of Iranian immigrants. These factors should be considered by policy makers and health care professionals when developing polices or interventions to improve the health of immigrants.
